# Insights on the Use of α-Lipoic Acid for Therapeutic Purposes

**DOI:** 10.3390/biom9080356

**Published:** 2019-08-09

**Authors:** Bahare Salehi, Yakup Berkay Yılmaz, Gizem Antika, Tugba Boyunegmez Tumer, Mohamad Fawzi Mahomoodally, Devina Lobine, Muhammad Akram, Muhammad Riaz, Esra Capanoglu, Farukh Sharopov, Natália Martins, William C. Cho, Javad Sharifi-Rad

**Affiliations:** 1 Student Research Committee, School of Medicine, Bam University of Medical Sciences, Bam 44340847, Iran; 2 Graduate Program of Biomolecular Sciences, Institute of Natural and Applied Sciences, Canakkale Onsekiz Mart University, Canakkale 17020, Turkey; 3 Department of Molecular Biology and Genetics, Faculty of Arts and Science, Canakkale Onsekiz Mart University, Canakkale 17020, Turkey; 4 Department of Health Sciences; Faculty of Science, University of Mauritius, Réduit 80837, Mauritius; 5 Department of Eastern Medicine, Government College University Faisalabad; Faisalabad 38000, Pakistan; 6 Department of Allied Health Sciences, Sargodha Medical College, University of Sargodha, Sargodha 40100, Pakistan; 7 Faculty of Chemical & Metallurgical Engineering, Food Engineering Department, Istanbul Technical University, Maslak 34469, Turkey; 8 Department of Pharmaceutical Technology, Avicenna Tajik State Medical University, Rudaki 139, Dushanbe 734003, Tajikistan; 9 Faculty of Medicine, University of Porto, Alameda Prof. Hernâni Monteiro, 4200-319 Porto, Portugal; 10 Institute for Research and Innovation in Health (i3S), University of Porto, 4200-135 Porto, Portugal; 11 Department of Clinical Oncology, Queen Elizabeth Hospital, 30 Gascoigne Road, Hong Kong; 12 Zabol Medicinal Plants Research Center, Zabol University of Medical Sciences, Zabol 61615-585, Iran

**Keywords:** α-lipoic acid, bioavailability, formulations, clinical trial, diabetic neuropathy, obesity, schizophrenia, sclerosis, pregnancy

## Abstract

α-lipoic acid (ALA, thioctic acid) is an organosulfur component produced from plants, animals, and humans. It has various properties, among them great antioxidant potential and is widely used as a racemic drug for diabetic polyneuropathy-associated pain and paresthesia. Naturally, ALA is located in mitochondria, where it is used as a cofactor for pyruvate dehydrogenase (PDH) and α-ketoglutarate dehydrogenase complexes. Despite its various potentials, ALA therapeutic efficacy is relatively low due to its pharmacokinetic profile. Data suggests that ALA has a short half-life and bioavailability (about 30%) triggered by its hepatic degradation, reduced solubility as well as instability in the stomach. However, the use of various innovative formulations has greatly improved ALA bioavailability. The R enantiomer of ALA shows better pharmacokinetic parameters, including increased bioavailability as compared to its S enantiomer. Indeed, the use of amphiphilic matrices has capability to improve ALA bioavailability and intestinal absorption. Also, ALA’s liquid formulations are associated with greater plasma concentration and bioavailability as compared to its solidified dosage form. Thus, improved formulations can increase both ALA absorption and bioavailability, leading to a raise in therapeutic efficacy. Interestingly, ALA bioavailability will be dependent on age, while no difference has been found for gender. The present review aims to provide an updated on studies from preclinical to clinical trials assessing ALA’s usages in diabetic patients with neuropathy, obesity, central nervous system-related diseases and abnormalities in pregnancy.

## 1. Introduction

α-lipoic acid (ALA), also known as 1,2-dithiolane-3-pentanoic acid or thioctic acid, is a compound commonly found in mitochondria, necessary for different enzymatic functions. ALA was isolated by Reed in 1951 [1] as an acetate replacing factor and its first clinical use dates from 1959 in the treatment of acute poisoning by *Amanita phalloides*, also known death cap (from mushrooms) [2].

**Figure 1:**
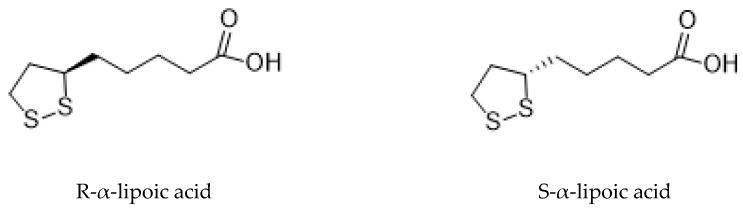
The chemical structure of optical isomers of ALA.

Briefly, ALA is an organosulfur compound produced from plants, animals, and humans and exists in nature. In the Krebs cycle, ALA plays important roles in various chemical reactions, acting as a cofactor for some enzymatic complexes involved in energy generation for the cell. It also forms covalent bonds with proteins and has therapeutic potential. It has a single chiral center and asymmetric carbon which results in two optical isomers: R- and S- lipoic acid (Figure 1) [3]. Thus, ALA has two enantiomeric forms, called S and R enantiomers, considered mirror images of each other. Both S and R enantiomers are present equally in ALA, being however the R isomeric form present naturally, while the S isomer is prepared through chemical processes. Foods are a natural source of the R enantiomer, naturally produced inside the living organisms forming covalent bonds with proteins. While ALA exists in nature as R enantiomer, synthetic supplementation consists of a racemic composition of R and S forms [4]. Although synthetized by the human body at low amount, the ALA quantities produced are not enough to fulfill the energy requirement of the cell. Thus, it is mostly obtained from diet, especially from meat and vegetables. Fruits are also a source of this acid [5]. 

On the other hand, ALA has numerous clinically valuable properties [5,6]. It acts as an enzymatic cofactor [7] and is also involved in glucose [8,9] and lipid [10] metabolism and manages gene transcription. ALA also acts as antioxidant because it not only improves but also restores the intrinsic antioxidant systems, and supports their production or cell accessibility [11,12,13]. It also efficiently removes heavy metals from blood stream, responsible for oxidative stress [11,13,14]. The most unique characteristic of ALA over other antioxidant substances is that it reacts as both lipid and water soluble compound [5,6]. There is no doubt that it is a strong antioxidant, but due to certain reasons its use for medicinal purposes is prohibited; however, in some states it is used as a supplement and in others as a remedy [5,6,15]. These restrictions are due to some endogenous characteristics of substance by itself, such as the changeableness due to the disclosing of dithiolane ring and the emergence of disulfide bond between molecules. Other properties that limit the oral use of ALA are its decreased ability to become dissolved in the gastrointestinal tract and increased rate of hepatic metabolism. In addition, besides it is widely known antioxidant potential, ALA has also many other functions, as it is its involvement in mitochondria producing energy, by acting as cofactor for various enzymes involved in metabolism [5].

Moreover, ALA plays a vital role in glucose humiliation during metabolism. For instance, ALA has been applied as a racemic medication for diabetic polyneuropathy-associated pain and paresthesia [16]. ALA has also an important function in energy transduction through mitochondria [6,17]. Two reduced or oxidized thiol groups are present in the small molecule of ALA. The oxidized form is known as ALA or simply as lipoic acid, while the reduced form is noted as dihydrolipoic acid (DHLA). ALA inactivates free radicals and the reduced form also interacts with reactive oxygen species (ROS) [8]. Naturally, ALA is found in mitochondria where it binds to the E2 subunit and is used as a cofactor for both pyruvate dehydrogenase (PDH) and α-ketoglutarate dehydrogenase complexes [18]. ALA is synthesized *de novo* at very small amounts in the body from cysteine and fatty acids, because of which there is a need to supplement it from exogenous sources [19].

ALA improves the glycemic control [6], alleviates diabetes mellitus (DM) complications [20,21] and even symptoms of peripheral neuropathy, at same time that effectively lessens the heavy metals toxicity [22].

### 1.1. Forms of Lipoic Acid

#### 1.1.1. R-α-Lipoic Acid

This isomer is present in nature, and is found in animals, plants and from human body. In nature, this is the form which ALA demonstrates its effects [23]. 

#### 1.1.2. S-α-Lipoic Acid

This type of isomer is not present in the nature. It may be obtained through many chemical procedures of thioctic acid and stops the important activities of R-ALA, e.g., their interaction with genes, enzymes and proteins [23].

ALA is found in many vegetables (spinach, broccoli, tomato, brussels sprouts, and rice bran), meats and entrails (e.g., liver and kidney) in lipoyllysine form (ALA with binding lysine residues). Moreover, ALA can also be synthesized by enzymatic reactions in mitochondria from octanoic acid and cysteine (as a sulfur donor) [24,25].

Both ALA and DHLA have a determinant role in oxidative metabolism [26]. For instance, it has been shown that ALA or its reduced form, DHLA have several positive health benefits, including as biological antioxidants, metal chelators and detoxification agents, being also able to reduce the oxidized forms of other antioxidant agents, including glutathione, vitamins C and E, and to modulate various signaling pathways, such as insulin and nuclear factor kappa B (NF-κB) [27]. It has also been used for age-associated cardiovascular, cognitive, and neuromuscular deficits [28,29,30], to reform endothelial dysfunction [31], to decrease oxidative stress [32] and to inhibit the formation of atherosclerosis plaque [33].

**Figure 2:**
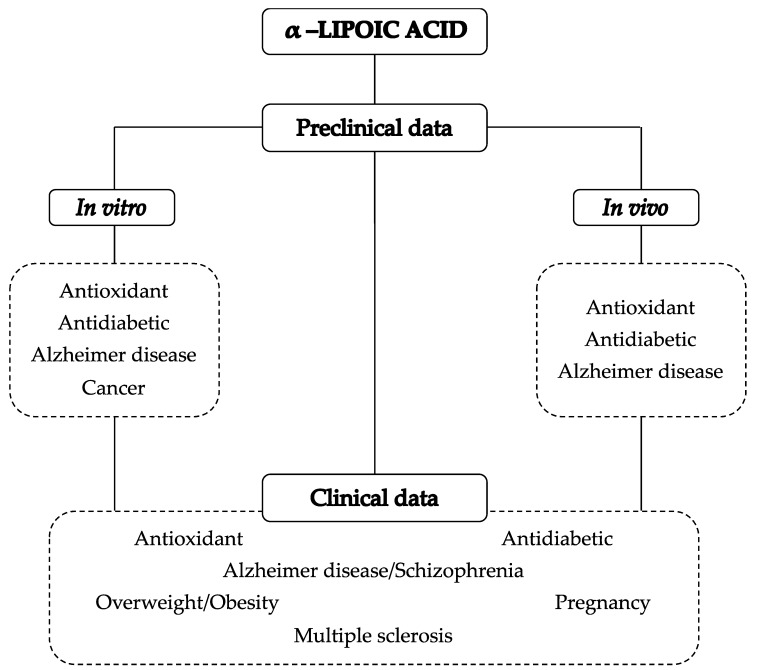
From preclinical to clinical effects of ALA.

In this sense, considering the potential therapeutic actions of ALA, we aim to focus on the preclinical and clinical studies assessing the ALA pharmacological effects, also considering the aspects related with its bioavailability (Figure 2).

## 2. Research Methodology

The search for the above-mentioned bioactive effects and clinical impact of ALA was performed in PubMed database, articles published in English were selected, from 2014 to 2019.

## 3. α-Lipoic Acid Pharmacological Activities: An Overview

Over the years, ALA has gained a considerable attention as a food additive with beneficial effects both in the treatment or management of several ailments [11,34,35]. ALA’s pharmacological effects are primarily related with its antioxidant activity, but ALA and DHLA have also demonstrated interesting cardiovascular, cognitive, anti-ageing, detoxifying, anti-inflammatory, anti-cancer, and neuroprotective properties [35].

### 3.1. α-Lipoic Acid Antioxidant Potential

There are vast literature data on ALA and DHLA antioxidant effects, namely acting as metal chelating agents, free radical scavengers, regenerator of endogenous antioxidants, such as glutathione, vitamins C and E and repair of oxidized damage [36]. The existence of thiol groups in ALA is responsible for its metal chelating abilities [14,35]. Moreover, it is able to increase the glutathione levels inside the cells, that chelate and excrete a wide variety of toxins, especially toxic metals from the body [35]. For instance, the study of Goralska et al. [37] showed that ALA administration led to a decrease in iron ions in epithelial cells. This change was associated with elevated cell resistance to hydrogen peroxide challenge, meaning that ALA exerts a direct impact in oxidative stress reduction [37]. Briefly, ALA is conceived as a biological antioxidant that is both water- and fat-soluble and is capable to neutralize ROS everywhere in the body, inside and outside the cells, and for this reason, ALA is being referred as the universal antioxidant [38,39,40].

### 3.2. α-Lipoic Acid Antidiabetic Potential

Among the metabolic disorders, diabetes mellitus (DM) represent a serious health problem, currently affecting approximately 422-million people worldwide [41]. It is designated by disturbances on carbohydrates, lipids, and proteins metabolism [42]. Also, it has been recognized as a major risk factor for the development of several human diseases, including atherosclerosis, hypertension, heart failure, myocardial infarction, neuropathic pain and even stroke [43]. Emerging evidences demonstrate that DM results from the excessive ROS generation and impairment of the antioxidant potential [44,45,46]. Several studies have highlighted the potential use of ALA in diabetes, due to its ability to increasing both sugar uptake in insulin-sensitive and insulin-resistant muscle tissues [4,47], and to stimulate the glucose uptake by the repartition of glucose transporters to the plasma membrane, and tyrosine phosphorylation of insulin receptor substrate-1 [9]. 

### 3.3. α-Lipoic Acid and Alzheimer’s Disease

Alzheimer’s disease (AD) is a neurological disease characterized by cognitive, functional, and behavioral alterations. Memory loss has been linked to the formation of beta-amyloid plaques and the uprise of tau in a pathological form in patients with AD [48,49]. Substantial evidences have supported the implication of oxidative stress in the pathogenesis of AD [50,51,52]. Non-steroidal anti-inflammatory drugs (NSAIDs) have been proposed for the therapy of neurodegenerative diseases, including AD. However, the prolonged NSAIDs administration results in gastrointestinal toxicity due cyclooxygenase (COX) inhibition [35,53]. To overcome this limitation, ALA has been selected based on the intended role of oxidative stress in the development of AD. 

In vitro investigations have indicated that ALA has neuroprotective effects on Aβ-mediated cytotoxicity [54,55,56], namely through defending cortical neurons from cytotoxicity induced by Aβ or H_2_O_2_ [57], partially attributed to the activation of PKB/Akt signaling pathway. Another study revealed that ALA has ability to effectively protect cultured hippocampal neurons against both Aβ peptide and Fe/H_2_O_2_ mediated toxicity [58]. 

Studies have also shown that ALA show anti-dementia or anti-AD properties by increasing acetylcholine (ACh) production through activation of choline acetyltransferase, which increases glucose absorption and, hence, supply more acetyl-CoA for ACh production [59]. Haugaard and Levin (2000) reported that DHLA significantly increased the activity of a purified preparation of choline acetyltransferase, and that its removal by dialysis from a partially purified of choline acetyltransferase led to a complete disappearance of enzyme activity and that its addition restores activity towards normal levels. The same finding was obtained when the experiments were repeated with extracts of rat brain and heart as well as rabbit bladder tissue. Thus, the authors concluded that it may act as a coenzyme in the choline acetyltransferase reaction [60]. 

On the other hand, inflammation has a key function in AD. It is engaged around amyloid plaques, surrounded by activated astrocytes and microglia, and is characterized by elevated levels of free radicals and pro-inflammatory cytokines [61], with TNF-α being considered an indicator from mild cognitive impairment to AD [59,62]. ALA has multiple and complex effects in this way, namely scavenging ROS, transition metal ions, increasing the levels of reduced glutathione [59,63], scavenging of lipid peroxidation products [62,64,65] and even acting on signal transduction pathways [63,66]. Similarly, Dinicola et al. [67] found that ALA downregulated the levels of the inflammatory cytokines IL-1B and IL-6 in SK-N-BE human neuroblastoma cells through DNA methylation-dependent modulation, paving the way for the impact of epigenetic mechanisms in AD control/prevention.

### 3.4. α-Lipoic Acid and Cancer 

An increasing body of literature highlight on the potential application of ALA in cancer therapy [68,69]. Cancer cells convert glucose preferentially to lactate for ATP generation, a phenomenon known as the Warburg effect or aerobic glycolysis. The persistent activation of aerobic glycolysis in cancerous cells lead to oncogenes activation or loss of tumor suppressors, thereby causing cancer progression. In this respect, the inhibition of aerobic cycle may contribute to anticancer effects [70,71]. Pyruvate dehydrogenase catalyzes pyruvate to acetyl CoA conversion, thus preventing lactate production. Feuerecker et al. investigated whether ALA is capable of activating pyruvate dehydrogenase in tumor cells. The results show that ALA inhibited cell proliferation, [18F]-FDG uptake and lactate formation and increased apoptosis in neuroblastoma cell lines Kelly, SK-N-SH, Neuro-2a and in the breast cancer cell line SkBr3. In the mouse xenograft model with subcutaneously SkBr3 cells, daily treatment with ALA has delayed tumor growth [72]. 

ALA suppressed thyroid cancer cell proliferation and growth through activation of AMPK and subsequent down-regulation of mTOR-S6 signaling pathway in BCPAP, HTH-83, CAL-62 and FTC-133 cells lines. In the same study, it was also found that ALA also significantly inhibited tumor growth in mouse xenograft model using BCPAP and FTC-133 cells [73]. In lung cancer cells, ALA inhibited cell proliferation through Grb2-mediated EGFR down-regulation [74]. 

Studies have also shown that ALA is able to generate ROS, which promote ALA-dependent cell death in lung cancer [75], breast cancer [76] and colon cancer [77,78], suggesting that it triggers the mitochondrial pathway of apoptosis in cancer cells. Recently, the effects of ALA on the migration and invasion of breast cancer cells were assessed [79]. The results have showed that ALA inhibited metastatic breast cancer cells migration and invasion, partly through ERK1/2 and AKT signaling. In summary, the scientific data show that ALA could be applied for cancer management and prevention. 

## 4. Preclinical Actability of α-Lipoic Acid

### 4.1. Anti-diabetic Properties of α-Lipoic Acid

As previously noted, ALA have shown to be useful for increasing sugar uptake in insulin-sensitive and insulin-resistant muscle tissues [4,47]. In addition, the triglycerides’ storage in the body led to type-2 DM progression. When activated, AMP-activated protein kinase (AMPK) increase sugar uptake, fatty acids oxidation and mitochondrial biogenesis. In obese rats, muscle AMPK levels are reduced. However, when these rats were submitted to ALA administration, a raise in insulin-stimulated glucose disposal in skeletal muscle and in the whole body was observed. ALA was also found to increase lipid oxidation and activated AMPK. These results suggest that ALA ameliorate insulin sensitivity through AMPK activation [80]. Konrad et al. [9] have demonstrated that ALA stimulates glucose uptake by the repartition of glucose transporters to the plasma membrane, and tyrosine phosphorylation of insulin receptor substrate-1. In a study carried out by Bitar et al. [81] it was found that the intake of 50 mg/kg for 30 days averted diabetes-mediated mitochondrial and endothelial dysfunction in rats, via a signal transduction pathway. It is known that in DM, the NO bioavailability is reduced through modulation of the endothelial nitric oxide synthase (eNOS) activity and oxidative stress [82]. In endothelial cells of aged rats, ALA intake resulted in a decrease in eNOS phosphorylation through Akt [83]. ALA is able to trigger Akt phosphorylation in human umbilical vascular endothelial cells and in THP-1 human monocyte cell lines [84,85]. These findings propose that the improved endothelial function due to ALA is partially ascribed to eNOS recoupling and increased NO bioavailability [82]. Thus, the use of ALA as an adjuvant in DM management is related to its capability to inhibit glycation which generates free radicals [82,86,87]. Overall, the information amassed herein indicated the potential therapeutic value of ALA for the treatment of DM.

### 4.2. α-Lipoic Acid and Alzheimer’s Disease

Given the above highlighted aspects on the use of ALA for neurodegenerative conditions, specifically in AD, Quinn et al. [88] assessed the effect of a diet supplemented with ALA on hippocampus-dependent memory of aged Tg2576 mice with AD. The authors found that ALA led to a marked improvement in learning and memory retention [88], and no significant differences were found in β-amyloid levels between ALA-treated and untreated Tg2576 mice [89]. 

### 4.3. α-Lipoic Acid and Pregnancy

Considering the promising antioxidant potential of ALA and its impact in multiple inflammatory conditions, recent evidences have increasingly highlighted its impact in physiological processes, such as pregnancy. Interestingly, Micili et al. [90] assessed the impact of ALA vaginal administration in female Wistar rats, namely testing its tissue distribution, impact on implantation process and effectiveness in contrasting induced preterm birth. Curiously, the authors found that vaginal ALA is well-absorbed and distributed, without affecting the implantation process and was even able to significantly revert mifepristone plus prostaglandin E2 effects, thus, delaying the delivery timing and decreasing the synthesis of mRNA and pro-inflammatory cytokines release [90].

## 5. Pharmacokinetics of α-Lipoic Acid

Although ALA has various biological activities, studies have reported a limited therapeutic efficacy due to its pharmacokinetic profile. Data suggests a short half-life and bioavailability of about 30% due to certain mechanisms, including hepatic degradation, reduced solubility as well as instability in the stomach [91]. However, this has been greatly improved through the use of various innovative formulations that directly increase ALA bioavailability.

### 5.1. Bioavailability of Lipoic Acid Through Food Sources 

In plasma and human cells, the amount of ALA is not enough to meet bodily needs, unless we take it through diet. The oral intake of ALA through diet has significantly increased its amount to fulfill the energy requirement of the body. Studies have reported a 40% increase in ALA absorption when ingested the mixture of both R and S isomers orally during fasting (empty stomach), while a 20% reduction of this acid occurs when taken through food sources. The efficacy of ALA R isomer shows more stability in plasma and is properly absorbed.

A study suggests that ALA bioavailability is greatly reduced after food intake and it has been recommended that ALA should be admitted at least 2 h after eating or if taken before; meal should be taken at least 30 min after ALA administration [92]. In addition, it has been suggested that acidic pH of the stomach is favorable for ALA absorption through the gastrum. Therefore, ALA supplements are preferably taken on an empty stomach to benefit of the acidic stomach pH. Moreover, it also reduces ALA competition with other nutrients for absorption [91]. Severe renal damages, as well as food intake, influences ALA pharmacokinetic parameters [93,94]. ALA can be taken through diet to fulfill its bodily requirements and can be received from natural sources. As previously highlighted, in animals, ALA is found in red meat, kidney, liver, and heart of animals, while in plants it is found abundantly in spinach, tomatoes, broccoli, brussels sprouts, garden peas, potatoes, and rice bran [11]. 

### 5.2. Lipoic Acid Absorption and Plasma Concentrations

It was observed that ALA is rapidly absorbed after oral ingestion of 50 to 600 mg thioctic acid. The time required to reach the maximum plasma concentrations was about 0.5 to 1 h. Moreover, the maximum plasma concentrations of R enantiomer were found to be 40 to 50 percent higher than the S enantiomer [95]. The R enantiomer of lipoic acid (RLA) was more rapidly absorbed through the intestine when given as an inclusion complex with γ-cyclodextrins. RLA/γ-cyclodextrins (CD) exhibit increased plasma exposure when compared to that of non-included R-lipoic acid. Moreover, the area under the plasma concentration-time curve (AUC) was 2.2 times higher than that of the non-included RLA orally administered, and 5.1 times higher when administered intraduodenally. Moreover, the absorption was not affected, even due to administration of an amylase inhibitor during the process and ligation of the bile duct [96]. In another work, carried out to assess the absorption of a racemic ALA formulation of 600 mg, it was found that ALA takes very little time to reach the maximum plasma concentrations of 6.86 ± 1.29 µg/mL.

It has been noted that plasma RLA concentration is higher than that of SLA at similar doses in humans [93]. A recent study performed in rats supports similar results for maximum plasma levels, as well as the AUC being almost 1.26 times higher for RLA compared to SLA [97]. Some researchers have reported that the rate of ALA uptake is not affected by the time the stomach is emptied, as found in a study conducted in insulin-dependent diabetic patients, in whom no particular effect on ALA bioavailability was observed [93].

### 5.3. Effect of Different Formulations on Lipoic acid Bioavailability

One study used 18 subjects of both genders, including 9 females and 9 males, and pharmacokinetic parameters were observed to assess the bioavailability of ALA in these subjects. Maximum concentration of ALA in plasma, time of maximum concentration beyond the terminal ALA half-life were observed and recorded in the study of Mignini et al. [98]. Since ALA is poorly soluble, lecithin has been used as an amphiphilic matrix to enhance its bioavailability. Tablets and soft gel capsules of ALA at a dose of 600 mg had the same bioavailability and other pharmacokinetic parameters, but were higher than those of the normally less soluble supplementation of ALA when administered to the human body [98]. It can be determined that the high bioavailability of ALA, as well as its homogenous release in vivo and high content can be increased by increasing its solubility through the use of amphiphilic matrices.

Similarly, another study determined the ALA bioavailability through different oral and intravenous (IV) formulations. The study used 200 mg ALA through the two administration routes to determine the pharmacokinetic parameters of ALA. The IV solution was given over 4 min, while the oral one consisted of 317.6 mg trometanlole salt, which corresponded to 200 mg of free ALA, 4 tablets of 50 mg and 1 tablet of 200 mg, given to 12 healthy subjects. The IV solution was the same as the oral solution. ALA could be detected for up to 2 h after IV drug administration and for up to 4 h after oral administration. However, it was determined that the maximum plasma concentration of ALA was greater through the IV route when compared to oral administration; in addition, the terminal half-lives for both routes were comparable. A previous study reported that the bioavailability of the R isomer was greater than that of the S isomer for all oral administrations, while the bioavailability of the R isomer was maximal through oral solution [93].

It has been suggested that ALA bioavailability is markedly increased when orally administered in the liquid form rather than a solid dosage form. Moreover, it presents prolonged stability, high plasma concentrations and accelerated absorption of ALA [5,7].

### 5.4. Age- and Gender-Dependent α-Lipoic Acid Bioavailability

Age greatly affects both ALA bioavailability and maximum plasma concentrations. Indeed, the bioavailability and peak plasma concentrations of ALA were found to be considerably higher in adults with mean age greater than 75 years as compared to young adults between the ages of 18 and 45 years. However, no significant variation in ALA bioavailability was found between males and females [17].

Another study demonstrated similar outcomes, with the exception that in tablet formulation (600 mg), the plasma concentrations of both ALA enantiomers were higher in females than males. At low concentration, no significant differences were stated [99].

## 6. α-Lipoic Acid in Clinical Trials

ALA has been extensively studied since the 1950’s, when its antioxidant properties were first discovered [100]. It has been demonstrated that ALA is effective in relieving some symptoms related to certain diseases, such as diabetes, age-related cardiovascular and neuromuscular defects, antipsychotic drugs-related weight gain and metabolic obesity [29,89,101,102]. Its potential effects on different types of diseases have drawn attention, since the results from studies were promising, namely in the field of neurodegenerative conditions [103]. In addition, the number of clinical trials increased to deepen knowledge on other ALA therapeutic properties and found hopeful effects. 

### 6.1. The Effects of α-Lipoic Acid on Diabetic Patients with Neuropathy

According to the World Health Organization (WHO), the number of diabetic individuals increased from 108 million in 1980 to 422 million in 2014; and in 2016, it was estimated that 1.6 million deaths were directly caused by diabetes [41,104]. Diabetes consists of a group of diseases caused by hyperglycemia and the effects of this condition fall into two major categories: macrovascular and microvascular complications. Retinopathy, nephropathy, and neuropathy are well-characterized microvascular complications, and the development of neuropathy is closely related to the extent and duration of hyperglycemia [105]. Diabetic neuropathy has also been recognized as a major cause of morbidity and mortality [106]. 

Effects of ALA on diabetes-associated neuropathy have been demonstrated by numerous clinical trials (Table 1). In the randomized, double-blind, placebo-controlled, multicenter, two-arm, parallel-group trial by Ziegler et al. [107], ALA was shown to be effective against mild-to-moderate diabetic sensorimotor polyneuropathy (DSPN). The treatment of diabetic patients with mild-to-moderate DSPN with 600 mg of ALA per day orally increased the neuropathy impairment score of lower limbs (NIS-LL) after four years. Increased NISS-LL scores greater than 2 were defined as a meaningful progression during the trial. All statistical analysis was measured in various subgroups of treatment groups. Among all subgroups, baseline subcategories with body mass index (BMI) lower than 30 kg/m^2^, patients with type 1 diabetes, clinically relevant smokers, and angiotensin-converting-enzyme (ACE) inhibitor-treated subgroups showed mean improvement (more than −1 point) in NIS-LL in ALA-treated group after 4 years. Subgroups including male patients older than 55 years-old, who have cardiovascular disease history and neuropathy more than 3 years with DSPN stage 2a showed a remarkable increase in NIS-LL in ALA-treated group when compared to placebo group. In this trial, it was shown that ALA might have the potential to prevent the neuropathic impairments progression with regular long-term administration. However, this trial was based on mild-to-moderate DSPN and may not be generalized. Further studies, including more severe stages of DSPN are needed to confirm these suggestions.

In a randomized, open-label trial, ALA activities were investigated in two consecutive phases [108]. Forty-five diabetes and symptomatic polyneuropathy patients were involved in phase 1 study. All participants received 600 mg ALA orally per day for 4 weeks and were instructed not to receive any drug that relieves neuropathic pain. Not all of the 45 patients completed the phase 1 because of patient withdrawal for personal reasons and use of prohibited drugs. After 4 weeks, patients with a Total Symptom Score (TSS) reduction more than 3 points were compared to their baseline value and continued to phase 2, where participants were randomly divided into two groups: one group continued ALA administration and the other one did not (control) for 16 weeks. The endpoint for phase 2 was the change in TSS, including burning and lancinating pain, paresthesias, and numbness. At the end of phase 2, TSS decreased in the ALA group, while no changes were stated to the control group. Furthermore, burning pain and paresthesias declined from randomization process to end of the trial; however, lancinating pain and numbness did not change in ALA-treated group in phase 2. Also, the use of analgesic rescue medication (for alleviating pain) was lower in the ALA-treated group. Thus, this trial showed that ALA improved neuropathic symptoms, while reducing the use of rescue medication in type 2 diabetes patients with symptomatic polyneuropathy.

In the trial by Sun et al. [102], a two-stage randomized, controlled study was conducted. In the first stage, 62 patients with early-stage diabetic nephropathy were separated into control and ALA-treated groups. Both groups continued to receive regular hypoglycemic therapy (routine treatment) and strict diet; however, they were not given ACE inhibitors. In the ALA-treated group, patients received 600 mg ALA per day intravenously for 2 weeks. In the second stage, 21 different patients were recruited for the study and divided into two groups: normoalbuminuria (urinary albumin excretion rates (UAER) lower than 30 mg/24 h) and microalbuminuria (UAER 30–300 mg/24 h). During the study, only one patient had side effects (mild nausea). Exosomes quality in urine samples were assessed by electron microscopy. Serum creatinine and malondialdehyde levels, as well as UAER were decreased in ALA group. Analysis of flow-mediated vasodilation (FMD) with several parameters showed a positive correlation only with superoxide dismutase (SOD) activity. Also, it was shown that expression levels of CD63-positive exosome were higher in ALA-treated group. This trial reported that in the early diabetic neuropathy, ALA could prevent the kidney from general oxidative stress in short-term use. 

Recently, a 40-day prospective, interventional trial by Agathos et al. [109] studied the action of ALA (600 mg/day, orally administered) on 72 diabetic patients with neuropathy, who were simultaneously taking their prescribed diabetic medications. Patients were scheduled to have 2 visits in 40 days: one at the beginning of the trial (baseline) and on the fortieth day (end day). In addition, blood samples were also collected to obtain baseline and second visit values. According to questionnaires results, neuropathy symptoms were reduced between the two visits. In laboratory results, mean fasting triglyceride levels were reduced significantly, whereas other parameters did not change between the two visits. Here, it was shown that ALA intake enhanced the quality of life of patients with diabetic neuropathy, reduced major symptoms and triglycerides levels.

### 6.2. Effects of α-Lipoic Acid in Overweight/Obese Patients

Obesity is a complex disorder, consisting in an abnormal fat storage that may lead to serious pathological diseases, not only in adults but also in children. WHO global estimates showed that the rate of obesity has nearly tripled between 1975 and 2016 [110]. Additionally, overweight (BMI 25-< 30) and obese (BMI ≥ 30) people have a significantly higher risk for increased mortality from diabetes, kidney and cardiovascular diseases and obesity-related cancers [111]. Moreover, the dysfunctional adipose tissue is a major merging factor between obesity and other secondary chronic diseases or carcinogenesis as a result of insulin resistance, chronic inflammation and altered adipokines secretion [112]. Thus, understanding the biology of weight regulation is crucial to discover effective interventional therapies for obesity and obesity-related disorders. In addition to the classical and known therapy for obesity, which consists of a combination of low-calorie diets and physical activity, researchers are increasingly exploiting new promising nutrient supplements to interrupt the cumulative risk of obesity [113]. Recently, the effects of ALA on weight control has been investigated by clinical trials, resulting in promising results worth to mention (Table 2).

In the study of Huerta et al. [114], 77 healthy overweight/obese women with BMI values between 27.5 and 40 kg/m^2^ were studied. All participants were randomly divided into 4 groups, treated with 1300 mg eicosapentaenoic acid (EPA) or 300 mg of ALA or 1300 mg of EPA plus 300 mg of ALA or placebo daily for 10 weeks. All individuals were adapted to 30% energy-restricted diet during this period. Accordingly, the ALA treated group showed significantly higher body weight loss and an important drop in leptin levels from the first week of treatment, despite no significant decrease in their resting metabolic rate was stated. A notable drop in triglycerides levels and diastolic blood pressure (DBP) was found in EPA plus ALA supplemented group. In general, all groups, except EPA supplemented one, had a significant reduction in leptin levels and marked improvements in insulin level during the oral glucose tolerance test (OGTT). No unfavorable effects were stated in the clinical trial period.

Huerta et al. [114] also investigated the potential relationship between circulating irisin and glucose metabolism and the effects of ALA or EPA on them. Irisin is a myokine; however, its role in obesity is not clear so far. A randomized, placebo-controlled, double-blind clinical trial with parallel design was conducted on 73 healthy overweight or obese women. The treatment groups design, supplementation doses for EPA, ALA, and the combination of EPA/ALA were identical to the above-mentioned research. Blood glucose levels demonstrated a significant decrease only in the control group and in the group of EPA and ALA combination. A considerably high significant reduction in body weight, hip circumference and fat mass were reported in ALA supplemented groups as compared to control and only EPA-treated groups. After weight loss, all groups showed decrease in irisin level; however, its concentration did not demonstrate significant differences between groups. Moreover, the analyses of changes in irisin level did not show significant correlation with weight loss, fat mass, and fat-free mass after 10 weeks of intervention, except for changes in insulin levels, which had positive relation. No substantial effects of ALA administration were obtained for irisin levels reduction in obese subjects. Therefore, more clinical interventions are needed in obese patients to clinically prove the effect of ALA in irisin production.

In another study, Li et al. [115] investigated the action of ALA therapy on body weight, waist circumference, and lipid metabolism in one hundred seventy overweight or obese patients (BMI ≥25 kg/m^2^). ALA group received orally 1200 mg ALA per day for 8 weeks, then after a 4-week washout intervention this group continued to receive placebo for 8 weeks. The exact opposite sequence of ALA and placebo interventions was used for placebo group. According to mixed model statistical analysis, ALA administration showed a significant body weight and waist circumference reduction. However, no significant differences found to leptin levels, lipid profile and adverse effects between the two groups. Only one female subject had severe nettle-rash in the ALA group. 

Hosseinpour-Arjmand et al. [116] assessed the effect of ALA on liver enzymes and inflammatory markers for non-alcoholic fatty liver disease (NAFLD), which is highly cooperating with the inflammatory components of obesity. A clinical trial was carried out on 45 obese patients with NAFLD, who received 1200 mg ALA plus 400 mg vitamin E or placebo involving 400 mg vitamin E per day for 12 weeks. ALA supplementation resulted in a notable increase in serum adiponectin levels and a reduction in IL-6 as well as insulin levels compared to placebo. Significant improvement in liver steatosis grade was detected for both ALA treated group (91.3%) and placebo group (54.5%) compared to their baseline value; however, changes were not statistically different between two groups. In the trial, the results indicated that 1200 mg of ALA supplementation per day was well-tolerated without any adverse effect. 

In the study of Escoté et al. [113], ALA administration was involved in another clinical trial to determine the relationship between fibroblast growth factor 21 (FGF21), which play a role as energy homeostasis regulator in metabolism and fatty acid profile. Fifty-seven overweight or obese women were administered with 1300 mg EPA or 300 mg ALA or 1300 mg EPA plus 300 mg ALA or placebo daily according to four different intervention groups with energy-restricted diet for 10 weeks. At the end of the trial, no significant relation was found between plasma FGF21 levels and weight loss or total fat mass for all experimental groups.

Romo-Hualde et al. [117] investigated the metabolomic changes occurred with the supplementation of 1300 mg EPA or 300 mg ALA or 1300 mg EPA plus 300 mg ALA or placebo per day on 67 healthy overweight/obese sedentary females by following an energy-restricted diet for 10 weeks. In this study, urine samples were used for pattern recognition and characteristic metabolites identification by principal component analysis and partial least squares-discriminant analysis. A higher reduction in BMI and fat mass were found for all ALA-supplemented groups compared to EPA-treated and placebo groups. Therefore, ALA administration may have beneficial effects on body weight reduction, however further studies are warranted.

### 6.3. Effects of α-Lipoic Acid on Patients with Schizophrenia 

Schizophrenia is a serious psychiatric and dysfunctional disorder that involves many symptoms. Hallucinations, delusions, and many neurocognitive deficits, including attention and memory loss, are the well-recognized symptoms [118]. The use of antipsychotic drugs relieves these symptoms to some extent [119], but they can also lead to several side effects, such as metabolic syndrome and weight gain [120,121]. Herein, trials describing the actions of ALA in schizophrenic patients were summarized (Table 3).

In the clinical trial of Kim et al. [101], 22 patients with schizophrenia were followed for 12 weeks in a double-blind, randomized placebo-controlled trial. Patients were divided into ALA-treated (n = 10) and placebo (n = 12) groups. Patients continued to use their antipsychotic drugs during the trial. ALA was administrated orally per day 30 min before each meal. The ALA dosage started at 1200 mg/day, then it was increased if the effect was not sufficient, or decreased if side effects were observed. The overall dose range for ALA was between 600–1800 mg/day. The primary outcomes were weight loss and decreased BMI in ALA-treated group. Plasma glucose and lipid profiles, as well as abdominal fat area by fat computed tomography scan were determined at the first and last day of the trial. Accordingly, only visceral fat area was found to be notably distinct between groups; however, no significant changes were detected in both sugar and fatty acid profiles. Thus, in this trial, it was shown that ALA seems to be effective against antipsychotic drugs-related weight gain. However, only a small number of patients was involved in this trial; so, studies with larger patient groups are needed to support these findings.

Vidović et al. [123] investigated the effects of ALA in 18 patients with schizophrenia to observe how plasma adiponectin levels and some metabolic risk factors change in a controlled clinical trial. ALA was administrated to all patients at 500 mg/day before breakfast for 3 months and patients were advised to continue to their usual dietary habits, antipsychotic drugs, and lifestyle. Blood sampling was handled at the beginning (baseline), in the middle and at the end of the trial. Fasting glucose, lipid status parameters, and liver enzymes were determined in serum samples. In addition, anthropometric measurements were analyzed including weight, height, waist circumference, and body fat. It is reported that after ALA treatment for three months, plasma adiponectin levels were increased significantly, whereas there were no remarkable changes in other factors. Furthermore, it was found a substantial reduction in fasting serum glucose and aspartate aminotransferase activity. Thus, this trial suggested that ALA may have a notable effect in the therapy of some metabolic risk factors in schizophrenia. Nevertheless, as this trial did not comprise a control group and open-label design, randomized controlled trials with larger patient groups are necessary to confirm the findings.

Recently, an open-label trial reported the effects of ALA in 10 patients with stable chronic schizophrenia [122]. The trial was conducted for 4 months with supplementation of 100 mg ALA/day with simultaneous use of prescribed antipsychotics. There were five visits and psychotic measurements were also obtained. At visits 1 and 5, neurocognitive assessments (Trail Making Test, Block Corsi Test, Subtest Digit Span, Category (Animal) Fluency and Controlled Oral Word Association Test, COWA (FAS test), and Rey Auditory Verbal Learning Test), collection of blood samples, measurement of abdominal circumference and body mass index (BMI) were carried out. At least a 25% decrease in negative/disorganization symptoms, including excitement (excitement, hostility, tension, grandiosity, and uncooperativeness), depression (depressive mood, guilt feelings, and motor retardation) and positive symptoms (unusual thought content, suspiciousness, and hallucinatory behavior) were observed between the first and last visits. Furthermore, there was a significant improvement in all neurocognitive tests, except for the Category (Animal) Fluency and the FAS test. There were no significant differences in abdominal circumference, BMI, complete blood count, levels of liver enzymes and other parameters. However, new trials with larger groups as well as randomized, double-blind and controlled design are needed to get reliable conclusions.

### 6.4. Effects of α-Lipoic Acid in Patients with Multiple Sclerosis

Multiple sclerosis is a known disabling disorder of the central nervous system (CNS) and attributed to multicentric inflammation and demyelination of CNS [124]. Inflammation in MS is developed by the invasion of T cells into the CNS, then they produce the matrix metalloproteinase-9 (MMP-9), which is an important protease correlated with MS relapse. Some studies proposed that ALA may be a useful drug for MS due to its inhibitory potential on T cells and inflammatory modulators migration [125,126]. 

MS with changing periods of the neurological disorder and recovery periods is described as a relapsing-remitting MS (RRMS), which is the most familiar type of MS. Additionally, if the neurological damage, such as axonal loss via inflammation-mediated demyelination increases in RRMS patients, this disease is often returned into a secondary-progressive disease course (SPMS) or even rarely to the primary progressive MS (PPMS). Thus, the attention on improving therapeutic agents to restrain progressive phases of MS is expanding in recent years [127]. The effects of ALA in this aspect were also investigated through a few number of clinical trials, summarized in Table 4. 

In the study of Khalili et al. [128] the anti-inflammatory effects of daily ALA consumption on RRMS patients were investigated. Forty-six patients were randomly separated into ALA group, which received 1200 mg ALA or placebo group, which received placebo per day for 12 weeks. A remarkable decrease in INF-γ, TGF-β, ICAM-1, and IL-4 levels was observed in ALA group when compared with the placebo group. However, no notable changes were stated in the levels of some cytokines, including TNF-α, IL-6, EDSS, and MMP-9 through ALA administration. Therefore, this study revealed preliminary supportive data on the anti-inflammatory effect of ALA on RRMS patients; however, further studies were needed with larger patients’ group to confirm these results.

The therapeutic effects of ALA on gait and balance deterioration, which are two critical symptoms for SPMS patients were investigated by Loy et al. [129]. The daily oral administration of 1200 mg ALA was examined and compared with placebo on 21 subjects during 2 years and the improvement in physical functions was assessed by Timed Up and Go (TUG) and quiet standing tasks in the particular periods. As a result, it was reported that ALA-treated patients with less disability showed a significantly better turning time in TUG-fast task, which measures the ability of patients how much walking quickly compared to the placebo group. Thus, this pilot study enabled an expanded clinical trial of ALA treatment for walking impairment in SPMS patients. 

Fiedler et al. [130] designed the first clinical trial to investigate the relationship between ALA and cAMP production and also the oral bioavailability of ALA by using healthy control, RRMS, and SPMS subjects. This study was completed by 57 subjects who received 1200 mg ALA orally for once. Blood probes were taken before and after 1, 2, 3, 4, 24 and 48 h from ALA administration for the cAMP measurements and ALA plasma levels by pharmacokinetic analysis. The reason for focusing on cAMP in MS patients was its inhibitory effects on proinflammatory cytokines expression and T-cells activation. Pharmacokinetics’ of plasma ALA concentration was compared between the healthy control, RRMS, and SPMS groups and showed no significant differences for half-life, Tmax, Cmax, volume of distribution, and oral clearance parameters. On the other hand, increased cAMP concentration was observed in healthy and SPMS subjects at 2 and 4 h of post-ALA treatment compared to baseline. Also, the ALA stimulatory effect on cAMP was analyzed by measuring plasma prostaglandin E2 (PGE2) levels, which is known as a cAMP stimulator and showed a significantly higher concentration in female healthy and SPMS subjects 4 h after ALA taking compared to RRMS subjects. In conclusion, these obtained data concluded that although the ALA stimulatory activity on cAMP production was divergent in RRMS patients, cAMP could be used as a biomarker to trace the medicinal actions of ALA in SPMS patients.

### 6.5. Effects of α-Lipoic Acid on Abnormalities in Pregnancy

Abnormalities in pregnancy, such as intrauterine bleeding or sub-chorionic hematomas are often associated with threatened miscarriage, especially during the first trimester of pregnancy. Sub-chorionic hematoma is a sonographically detected anechoic area with a falciform shape that increases the risk of spontaneous abortion [131]. Placental dysfunction, insufficient angiogenesis, and chronic inflammation are underlying causes of early pregnancy bleeding, and these factors can even result in preterm labor or perinatal mortality [132]. Progesterone has a significant role both in maturation of fetus and cytokine balance. Therefore, administration of progestogens is mostly used to prevent threatened miscarriage [133]. On the other hand, ALA treatment has been recently studied by some clinical trials to explain its efficacy in preventing miscarriage (Table 5).

A preliminary randomized clinical trial was done by Porcora et al. [134] to assess the supportive action of ALA with progesterone therapy in the recovery of sub-chorionic hematomas on 16 patients with threatened miscarriage. The subjects were between 6th and 13th week of pregnancy with pelvic pain, vaginal bleeding, and sub-chorionic hematomas. They were randomly divided into two groups. Accordingly, both control and case groups were administered with 400 mg progesterone/day in the form of vaginal suppositories. Besides, case group was additionally treated with 600 mg ALA/day until complete resolution of the clinical picture. Medical examinations of all subjects were carried out after a week from the enrollment, and later every fifteen days until the symptoms disappeared. During treatment, undesirable effects were not reported either in the mother or fetus. Earlier and better improvements in the symptoms of sub-chorionic hematomas were detected in the ALA plus progesterone treatment group when compared to progesterone alone. However, the change of soft uterus, which is a symptom of threatened miscarriage was not statistically significant in both groups. This study may suggest that ALA may be beneficial for health of both mother and fetus in case of threatened miscarriage.

A pilot, randomized, placebo-controlled, parallel group and monocenter study of Grandi et al. [135] studied the anti-inflammatory activities of ALA on cervical inflammation and shortening after primary tocolysis. Thirty-two women with a singleton pregnancy, at gestational age ranging 24–30 weeks and hospitalized for a first preterm labor episode were randomly recruited for the intake of 400 mg/day of ALA in the form of vaginal hard tablets (active ingredient 10 mg) or placebo before sleep for 30 days. Their cervical-vaginal fluids were obtained by cervical swab to quantify the levels of pro-inflammatory cytokines before and after treatment in both groups. Moreover, cervical length tracing [122], whose shortening is a clue for preterm birth, was achieved by transvaginal ultrasound method before and after treatment. These analyses showed a notable enhance in both IL4 and IL10 levels by vaginal ALA treatment compared to placebo, while no remarkable changes were found to proinflammatory cytokines ratio between groups. Another important effect of ALA was observed in CL measurements. Accordingly, it was reported that the shortening of the cervix was restricted more effectively in vaginal ALA than in control group. Overall, these results encourage larger randomized and controlled clinical trials on this topic.

In a study, Costantino et al. [136] compared the actions of vaginal ALA or progesterone treatment in sub-chorionic hematoma resorption in 62 pregnant women with threatened miscarriage. Two treatment groups (1:1 ratio) were defined: one received 400 mg of vaginal progesterone (vaginal soft gel) daily or 10 mg of vaginal ALA (vaginal capsule) and one control group without treatment (upon their requests) during 60 days. The development of sub-chorionic hematoma was controlled by a vaginal ultrasound scan after 20 days and 60 days and no adverse effects on the fetus were recorded. Significant improvements and a smaller number of miscarriages were observed for the sub-chorionic hematoma resorption in the ALA-treated group, compared to progesterone group. However, no remarkable variations were recorded in pelvic pain and vaginal bleeding values in any of the groups. Therefore, these primary data support that ALA can be an effective medical route for the treatment of patients with threatened miscarriage; nevertheless, more studies are needed to confirm this usage. 

### 6.6. Other Trials

There are small numbers of trials assessing the effects of ALA in organ transplantation and in case of chemotherapy. 

In the work of Ambrosi et al. [137], the effects of ALA against ischemia-reperfusion injury (IRI) which occurred after simultaneous kidney-pancreas transplantation were investigated. Twenty-six patients with diabetic polyneuropathy were separated into three groups: no treatment, donor and recipient ALA-treated and only recipient ALA-treated groups. 600 mg ALA were administrated to ALA-treated groups before surgery. The aims of including two treated groups (recipient treated—recipient and donor treated) was to figure out the influence of produced ROS at the different stages of IRI. Pancreatic and kidney biopsies were done at the end of the surgery to perform polymerase chain reaction (RT-PCR); however, the amount of biopsied tissue was not enough to perform immunohistochemical staining. Blood probes were obtained before and after surgery. The levels of serum inflammatory cytokines and other measurements were carried out. Low levels of TNFα and C3 in kidneys, and high levels of heme oxygenase-1 (HMOX-1) and C3 in the pancreatic biopsies were reported. Furthermore, the decrease in IL-8, IL-6, secretory leukocyte protease inhibitor, and regenerating islet-derived protein 3 β/pancreatitis-associated protein levels were recorded in the donor-recipient treated group. This study showed that ALA can be effective in reducing inflammatory markers, kidney dysfunction and clinical pancreatitis in post-transplant patients. However, there are few studies including organ transplantation. Thus, there is a need for further randomized, placebo-controlled studies to obtain more reliable results on ALA effectiveness.

Recently, Casciato et al. [138] carried out a clinical trial in 40 liver transplant patients. Patients were divided into two groups: ALA-treated and placebo. 600 mg of ALA was administrated to patients in the donor portal vein immediately before the cold ischemia time; then, another 600 mg of ALA was administrated 15 min prior to the reperfusion. Liver tissue samples were collected from the donor and from patient 2 h after reperfusion. Blood probes were taken before and at the end of surgery. Samples were collected 5 times in a month after surgery. Biochemical liver parameters were also measured. High levels of hypoxia-inducible factor-1 (HIF-1α) and prolyl hydroxylase-2 (PHD2) were reported in liver biopsies for ALA-treated group, whereas no significant differences were stated to other hypoxia-related parameters. In addition, it was shown that baculoviral IAP repeat containing 2 (Birc2) transcription levels were also higher in the ALA-treated group. Also, the plasma levels of alarmins were lower in ALA-treated patients. Overall, these results suggest that the use of ALA in liver transplantation is safe as it can be protective against hypoxia and oxidative stress by inducing changes in the gene expressions at the mRNA levels.

Finally, Guo et al. [139] assessed the effect of ALA on prevention of chemotherapy-induced peripheral neuropathy. Forty cancer patients completed the trial. They were divided into two groups: ALA-treated and placebo. Further, these groups were separated to three groups according to their platinum exposure levels. 1800 mg of ALA or placebo were administrated orally every day, except during the period 2 days before to 4 days after administration of each dose of platinum to avoid potential interference with platinum’s antitumor effects. Neuropathic symptoms were measured at baseline and then at 24, 36, and 48 weeks of treatment. Besides, Brief Pain Inventory (BPI) Partial Forms (it included three-item questionnaires) were given to patients to learn their pain symptoms during the trial. No remarkable changes were recorded between groups for all parameters measured. It was concluded that ALA was ineffective against the neurotoxicity induced by the action of oxaliplatin or cisplatin, so that it is necessary to conduct future studies.

## 7. Conclusions

ALA has various benefits, including antioxidant potential; however, it has been shown that the therapeutic efficacy of ALA is restricted because of limitations related with its pharmacokinetic profile. Data shows a short half-life and bioavailability of about 30% of ALA due to mechanisms involving hepatic degradation, reduced ALA solubility as well as instability in the stomach. However, the use of various innovative formulations has proved to be effective in enhancing ALA bioavailability. It has been shown through studies that ALA bioavailability is enhanced through the use of amphiphilic matrices, able to enhance its solubility and absorption in the intestine. Moreover, ALA liquid formulations show greater plasma concentrations and bioavailability as compared to solid dosages. Moreover, age also affects ALA bioavailability, while gender shows insignificant differences. Thus, improved formulations that can enhance ALA absorption will markedly improve ALA bioavailability, ultimately leading to an improved therapeutic efficacy.

When looking at data from clinical trials, ALA has revealed to be efficient in particular diseases and conditions, including diabetic neuropathy, obesity, schizophrenia, MS, abnormalities in pregnancy and organ transplantation, with no or minor adverse effects. ALA seems to be also a promising agent to improve quality of life, as well as neuropathic symptoms and even to reduce the use of rescue drugs, which are commonly used by patients with diabetic neuropathy. Moreover, it has the potential to improve the lipid metabolism and promote weight reduction in obese patients, besides to alleviate CNS-related diseases (Schizophrenia and MS) symptoms. ALA may also decrease body mass gain caused by the application of prescribed antipsychotic agents, as well as some metabolic risk factors in patients with schizophrenia. In patients with MS, ALA has some positive outcomes, especially in the enhancement of walking and balance disabilities, while decreasing the levels of some proinflammatory factors related to MS progression. Therefore, the clinical trials assessing the ALA effects show its ability to alleviate some symptoms, commonly found in CNS diseases, with highly promising results. In case of threatened pregnancies, ALA demonstrated beneficial effects on the enhancement of sub-chronic hematoma symptoms, as well as positive results in miscarriage prevention, with no adverse effects. ALA is also effective in organ transplantation patients by reducing the levels of inflammatory factors and exerting protective effects against hypoxia and oxidative stress, whereas in case of neurotoxicity caused by cytotoxic chemotherapy medication ALA did not represent a protective function. Taken all together, ALA may be classified as one of the candidate molecules for prevention or slowing down some conditions associated with several diseases’ progression. However, more controlled and robust clinical trials must be designed for investigating ALA therapeutic effects.

## Figures and Tables

**Table 1:** Effects of ALA in diabetic patients with neuropathy.

Patients (n)	Design	Treatment	Key Effects	References
Diabetic patients with mild-to-moderate polyneuropathy **Age range:** n.s. **n** = 429	Clinical trial Randomized Double-blind Placebo-controlled Multicenter Two-arm	600 mg/day ALA or placebo, orally **Duration:** 4 years	- Prevention of neuropathic improvements progression with regular and long-term administration	[107]
Type 2 diabetic patients with symptomatic polyneuropathy **Age range:** n.s. **n** = 45	Clinical trial Randomized Withdrawal Open-label study	600 mg ALA 3 times per day in phase 1, orally 600 mg ALA daily or ALA withdrawal in phase 2, orally **Duration:** 4 weeks (phase 1) 16 weeks (phase 2)	- Phase 1: Total Symptom Score (TSS) decreased - Phase 2: TSS decreased in ALA-treated group and improved neuropathic symptoms	[108]
Diabetic patients with early nephropathy **Age range:** n.s. **n** = 62	Clinical trial Randomized Controlled	600 mg/day ALA, intravenously with routine treatment or routine treatment (control group) **Duration:** 8 weeks	- Decline in urinary albumin excretion rates, serum creatinine and malonaldehyde - Increased plasma SOD activity and improved endothelium-dependent flow mediated vasodilation flexibility	[102]
Diabetic patients with neuropathy **Age range:** 18–75 **n** = 72	Clinical trial Clinical report Interventional study	600 mg/day ALA, orally **Duration:** 40 days	- Reduction in neuropathic symptoms and triglycerides levels	[109]

**Table 2:** Effects of ALA in overweight/obese patients.

Patients (n)	Design	Treatment	Key Effects	References
Overweight/ obese women **Age range:** 20–50 **n** = 77	Clinical trial Randomized Double-blind Placebo-Controlled Parallel design	1300 mg/day EPA or 300 mg/day ALA or both 1300 mg/day EPA + 300 mg/day ALA or placebo, orally 30% energy-restricted diet **Duration:** 10 weeks	- Significantly higher body weight loss in ALA treated groups - Significantly attenuated decrease in leptin levels in ALA treated groups during weight loss	[114]
Overweight/ obese women **Age range:** 20–50 **n** = 73	Clinical trial Randomized Double-blind Placebo-controlled Parallel design	1300 mg/day EPA or 300 mg/day ALA or both 1300 mg/day EPA + 300 mg/day ALA or placebo 30% energy-restricted diet **Duration:** 10 weeks	- A high reduction in body weight, BMI and fat mass was stated in ALA treated groups - Significant reduction in glucose levels for only control group and EPA + ALA group - No significant differences in irisin changes between groups	[114]
Overweight or obese patients **Age range:** 38–47 **n** = 170	Clinical trial Single-center Randomized Double-blind Crossover controlled	1200 mg/day ALA or placebo, orally **Duration:** 8 weeks	- Significant reduction in body weight and waist circumference	[115]
Obese patients with non-alcoholic fatty liver disease (NAFLD) **Age range:** 20–50 **n** = 45	Clinical trial Randomized Double-blind Placebo-controlled	1200 mg/day ALA + 400 mg/day vitamin E or vitamin E (placebo), orally **Duration:** 12 weeks	- Significant improvement in serum adiponectin and IL-6 levels	[116]
Overweight/obese women **Age range:** not specified **n** = 57	Clinical trial Randomized Double-blind Placebo-controlled	300 mg/day ALA or 1300 mg/day EPA or 1300 mg/day EPA + 300 mg/day ALA or placebo, orally Hypocaloric diet **Duration:** 10 weeks	- A significant reduction in the circulating levels of saturated fatty acid and total n-6-PUFAs	[113]
Overweight/ obese sedentary females **Age range:** n.s. **n** = 65	Clinical trial Randomized Double-blind Placebo-controlled	300 mg/day ALA or 1300 mg/day EPA or 1300 mg/day EPA + 300 mg/day ALA or placebo, orally Energy restricted diet **Duration:** 10 weeks	- Significant reduction in BMI and fat mass in ALA treated groups	[117]

**Table 3:** Effects of ALA in schizophrenic patients.

Patients (n)	Design	Treatment	Key Effects	References
Schizophrenia with antipsychotic induced weight gain **Age range:** n.s **n** = 15	Clinical trial Randomized Double-blind Placebo-controlled	600–1800 mg/day ALA or placebo, orally **Duration:** 12 weeks	- Reduction in body weight and BMI - Significantly reduced visceral fat areas - No severe side effects except gastrointestinal symptoms and mild dermatologic symptoms	[101]
Schizophrenia **Age range:** 18–60 **n** = 10	Clinical trial Open-Label Trial	100 mg/day ALA, orally **Duration:** 4 months	- Significant improvement in neurocognitive parameters - No significant differences in BMI, abdominal circumference, blood count and liver enzymes	[122]
Schizophrenia **Age range:** 25–60 **n** = 18	Clinical trial Controlled	500 mg/day ALA, orally **Duration:** 3 months	- Significant increase in plasma adiponectin levels - Decrease in fasting glucose and aspartate aminotransferase activity	[123]

**Table 4:** Effects of ALA in patients with multiple sclerosis (MS).

Patients (n)	Design	Treatment	Key Effects	References
Relapsing-remitting MS **Age range:** 18–50 **n** = 52	Clinical trial Randomized Double-blind Placebo-controlled	1200 mg/day ALA or placebo, orally **Duration:** 12 weeks	- Significant reduction in serum levels of INF-γ, ICAM-1 TGF-β and IL-4 - No significant changes in TNF-α, IL-6, EDSS and MMP-9 levels	[128]
Secondary progressive multiple sclerosis (SPMS) **Age range:** 40–70 **n** = 21	Clinical trial Randomized Double-blind Placebo-controlled Pilot study	1200 mg/day ALA or placebo, orally **Duration:** 2 years	- Significant improvements in walking performance in patients	[129]
Relapsing and remitting MS (RRMS), secondary progressive MS (SPMS) **Age range:** age ≥ 18 **n** = 57	Clinical trial Controlled	1200 mg racemic ALA once **Duration:** 48 h	- Increased cAMP at 2 and 4 h of ALA treatment in healthy and SPMS patients - Decrease cAMP in RRMS patients	[130]

**Table 5:** Effects of ALA on abnormalities in pregnancy.

Patients (n)	Design	Treatment	Key Effects	References
Threatened miscarriage and subchorionic hematoma **Age range:** 20–40 **n** = 16	Preliminary Clinical trial Randomized	600 mg/day ALA + 400 mg/day Progesterone or 400 mg/day Progesterone (control group), orally **Duration:** until complete resolution of the clinical picture	- Effective determination in major signs of threatened miscarriage in ALA-treated group - Significant improvements for hematoma resorption in ALA-treated group - No adverse effects on mother or fetus	[134]
Singleton pregnancy, at a gestational age ranging 24–30 weeks, hospitalized for a first preterm labor episode **Age range:** n.s. **n** = 32	Clinical trial Randomized Placebo-controlled Pilot study	400 mg/day ALA (active ingredient 10 mg) or placebo, vaginal tablets **Duration:** 30 days	- Significant increase in anti-inflammatory interleukins in the cervical vaginal liquids of undelivered women after a preterm labor episode	[135]
Threatened miscarriage **Age range:** 24–40 **n** = 62	Clinical trial Randomized Controlled	10 mg/day ALA (vaginal capsule) or 400 mg/day progesterone (vaginal soft gel) or placebo **Duration:** 60 days	- quick reabsorption of sub-chorionic hematoma in ALA-treated group - Smaller number of miscarriages in ALA-treated group	[136]
